# DDX39 Overexpression Predicts a Poor Prognosis and Promotes Aggressiveness of Melanoma by Cooperating With SNAIL

**DOI:** 10.3389/fonc.2020.01261

**Published:** 2020-08-12

**Authors:** Chengjuan Xing, Hui Tian, Yini Zhang, Kun Guo, Ying Tang, Qimin Wang, Li Lv, Lifen Wang

**Affiliations:** ^1^Department of Pathology, The Second Affiliated Hospital of Dalian Medical University, Dalian, China; ^2^Department of Emergency Medicine, Dalian Municipal Central Hospital Affiliated of Dalian Medical University, Dalian, China

**Keywords:** melanoma, DDX39, SNAIL, aggressiveness, prognosis

## Abstract

This study aimed to investigate the prognostic value and molecular mechanism of DDX39 and its effector SNAIL in melanoma. First, overexpression of DDX39 in melanoma, which was identified by database analysis, was further validated in patient tissues. Cell growth, cell cycle, cell migration, and cell invasion assays were then performed to evaluate the effects of downregulated DDX39 on the melanoma cell proliferation and aggressiveness. The same approaches were also used to reveal the cooperation of the transcription factor SNAIL with DDX39 to promote the aggressiveness of melanoma cells. We found that the expression of DDX39 was significantly upregulated in melanoma tissue compared to pigmented nevus tissue, and it was positively correlated with the clinical stage defined by the American Joint Committee on Cancer (AJCC) and the prognosis. Downregulation of DDX39 in melanoma cells was found to significantly inhibit cell proliferation, increase G2/M cell cycle arrest, enhance caspase-mediated cell apoptosis, and suppress cell invasion and migration. In addition, we demonstrated that the overexpression of SNAIL could restore the cell growth and aggressiveness impaired by DDX39 RNA interference. Immunohistochemical staining also showed a positive correlation between DDX39 overexpression and SNAIL overexpression in melanoma tissues, suggesting that SNAIL is one of the effectors activated by DDX39. In summary, the overexpression of DDX39 and SNAIL was positively related to the poor prognosis of melanoma patients and the increased aggressiveness of melanoma cells. Our study provides valuable evidence regarding the prognostic value of DDX39 and SNAIL as well as their potential as novel therapeutic targets for treating melanoma patients.

## Introduction

Melanoma is a malignant tumor originating from the skin or mucosal pigmented cells and is the leading cause of death from malignant skin tumors; the worldwide morbidity of melanoma is increasing by 3–8% per year (1). The American Cancer Society has estimated that around 100,350 new melanomas will be diagnosed, and about 6,850 people in the United States are expected to die from melanoma in 2020 (https://www.cancer.org/cancer/melanoma-skin-cancer/about/key-statistics.html). According to the site of the disease, melanoma can be divided into cutaneous melanoma, mucosal melanoma, and choroidal melanoma. Among them, cutaneous melanoma is the most common subtype. In western countries, cutaneous melanoma is mostly associated with sunlight exposure; while in China, most cases occur at the extremities. Melanoma, characterized by occult onset, rapid progression, and low survival rates in advanced patients, is insensitive to radiotherapy, and chemotherapy, making surgery the most effective treatment (2). Since 2011, Vemurafenib (BRAF inhibitor), Trametinib (MEK inhibitor), Ipilimumab (cytotoxic T-lymphocyte-associated protein 4 monoclonal antibody), as well as Nivolumab and Pembrolizumab (programmed cell death protein 1 monoclonal antibodies) have been successively approved by the US Federal Drug Administration for melanoma treatment. However, the current clinical data show that only a small number of patients benefit from these novel therapeutics. For example, Vemurafenib, the inhibitor designed against mutations in the BRAF V600 gene, has an objective response rate of 52% in patients with targeted variants (3). However, the mutation rate of the BRAF V600 gene of melanoma patients in China is only 25%, which is lower than the rate of 50–60% found in western countries (4). The mechanism of melanoma pathogenesis is complex, and further exploration is needed to identify more effective and specific therapeutic targets for treatment. Retrospective studies have shown that most patients with melanoma, especially those with acral melanoma, had a history of pigmented nevus for several years or even two to three decades (5). However, when rapid swollen pigmented bulges were found to have irregular edges as well as blisters and poor-healing ulcers on the surface, pathology tests revealed the presence of melanoma. Hence, studying the gene expression discrepancy between pigmented nevi and melanoma would be helpful to identify genes involved in melanoma progressiveness.

In the present study, by analyzing the Affymetrix expression profile in the Gene Expression Omnibus (GEO) database GSE 46517, we first identified DDX39 (6), a member of the DEAD box RNA helicase family, as an overexpressed gene in melanoma but not in pigmented nevi tissues. We also examined the overexpression of DDX39 protein in melanoma patients and its correlation with clinical features and a poor prognosis. Next, we studied the impact of DDX39 on the *in vitro* proliferation, migration, and invasion of melanoma cells. Finally, we investigated the role of the transcription factor SNAIL in the DDX39 pathway on activation of the aggressiveness of melanoma cells.

## Materials and Methods

### Bioinformatics Analysis

The GEO database (https://www.ncbi.nlm.nih.gov/geo/) is a public repository that stores high-throughput gene expression datasets. In this study, the dataset GSE46517, containing Affymetrix expression profiling, was explored to identify differences in the mRNA levels between 31 primary melanoma and 9 melanocytic nevus tissue samples. The Affy and Limma packages in R software were used to perform the standardization and pairwise *T-*test analysis of these data. All of the analytical results were then filtered according to the criteria of *P* < 0.05 and fold change ≥ 1.5, and the differentially expressed genes were identified after annotation.

### Patients and Clinical Data Collection

The melanoma samples and the related clinical information were obtained from patients undergoing skin melanoma surgery from January 26, 2010, to August 29, 2018, in the Second Affiliated Hospital of Dalian Medical University. None of the patients had received radiotherapy, chemotherapy, or other anticancer treatments before surgery. The patients included 27 males and 24 females aged between 17 and 85 years, with an average age of 37 years. All patients were followed up by text or phone, or as outpatients, and 8 of them were lost to follow-up. The longest follow-up time was 122 months, and the shortest follow-up time was 1 month. The follow-up deadline was May 31, 2019. The overall survival time was counted from the day of operation to the end of follow-up. The disease-free survival time was counted from the date of surgery to the time of disease recurrence or metastasis. This study was approved by the Ethics Committee of the Second Affiliated Hospital of Dalian Medical University and was performed in accordance with the guidelines of the Declaration of Helsinki (No. BBMCEC2012063). All patients provided written informed consent to participate in this study.

### Immunohistochemistry

The primary melanoma tissues from these 51 patients and the matched recurrent and metastatic tissues from 15 of them were collected. Briefly, the slides of paraffin-embedded tissues were placed in antigen repair solution, followed by high-pressure thermal repair. Then, the sections were incubated with primary antibody against DDX39 (1:100, ab176348, Abcam) or SNAIL (1:100, ab53519, Abcam) overnight at 4°C after being blocked in goat serum. The slides were further washed and incubated with biotinylated secondary antibody and horseradish peroxidase-labeled streptavidin working solution (Zsbio, Beijing, China) at room temperature for 20 min. The stained tissues were finally developed in 3,3′-diaminobenzidine colorant (Zsbio, Beijing, China), and the slides were counterstained with hematoxylin. The immunohistochemical staining was independently evaluated by two pathologists and quantified according to the scoring system described previously (7, 8).

### Cell Culture and Transduction

The human melanoma cell lines A375 and SK-MEL28 were obtained from the Cell Resource Center, Shanghai Institute of Biochemistry and Cell Biology at the Chinese Academy of Sciences (Shanghai, China). Cells were cultured at the appropriate density in Dulbecco's modified Eagle medium supplemented with 10% (v/v) fetal bovine serum (FBS) and 1% antibiotics (100 μg/mL streptomycin and 100 U/mL penicillin) in a humidified incubator containing 5% CO_2_ at 37°C. The recombinant lentivirus carried short hairpin RNA (shRNA) against DDX39A, the overexpression cassette of SNAIL; and the control vectors were designed, constructed, and titered by the Shanghai GeneChem Company (http://www.genechem.com.cn/). The lentiviral vector used for shRNA carried the gene encoding green fluorescent protein, while the lentiviral vector used for overexpression carried the gene encoding red fluorescent protein. The multiplicity of infection of transduction was optimized to ensure that the observed fluorescence positivity was over 80%.

### Real-Time Quantitative Polymerase Chain Reaction (qPCR)

Total RNA was isolated from whole-cell lysates by TRIzol, according to the manufacturer's instructions (Sigma, USA). RNA was reverse-transcribed by an M-MLV kit (Promega, Madison, WI, USA), and the synthesized cDNA was subjected to qPCR using the SYBR® Green qPCR SuperMix kit (TaKaRaBio, Dalian, China), according to the manufacturer's instructions. Glyceraldehyde 3-phosphate dehydrogenase (GAPDH) was used as an internal control. The melting curves and E = 2^−ΔΔCt^ algorithm were analyzed by LightCycler software (Roche Diagnostics).

### Western Blot Analysis

Total protein of the cell lysate in each group was extracted using NP-40 lysis buffer (Beyotime Biotechnology, Shanghai, China). The protein concentration was determined using a bicinchoninic acid protein concentration quantification kit (Beyotime Biotechnology). Denatured proteins were separated by sodium dodecyl sulfate–polyacrylamide gel electrophoresis and transferred to a polyvinylidene difluoride membrane. The membrane was blocked with 5% non-fat dry milk in Tris-buffered saline containing 0.05% Tween 20 and probed with primary antibodies overnight at 4°C. Following washing, the membrane was incubated with goat horseradish peroxidase-conjugated secondary antibodies at room temperature for 2 h (1:2,000 dilution, Santa Cruz Biotechnology, Santa Cruz, CA, USA). The reactions were detected by using Enhanced Chemiluminescence Western Blotting Substrate (Pierce, ThermoFisher Scientific, Shanghai, China). The detected bands were visualized via exposure to an x-ray beam in a dark room and semi-quantified via gray-scale analysis.

### Cell Proliferation Assay

The MTT assay was performed in triplicate daily for 5 days. Cells in the log phase of growth were seeded into 96-well plates at a density of 2.5 × 10^3^/well in 100 μL of complete medium. After 20 h, 20 μL of MTT (5 mg/mL) (Sigma-Aldrich, St. Louis, MO, USA) was added to each well, followed by incubation at 37°C in 5% CO_2_ for 4 h. Subsequently, 100 μL of dimethyl sulfoxide (Sigma-Aldrich) was added, and the plate was shaken for 20 min at room temperature to dissolve the formazan crystals. The optical density (absorbance units) was read at a wavelength of 490 nm on a microplate reader (Tecan infinite M2009PR, Männedorf, Switzerland). The growth inhibition curve was generated using Microsoft Excel (Seattle, WA, USA).

Cell proliferation analysis was performed using a Celigo Imaging cytometer. Cells in the log phase of growth were plated into 96-well plates at a density of 10^3^ cells/well in 100 μL of complete medium and incubated for 24 h. Beginning on day 2, the cells were imaged and counted daily using the Celigo Cell Counting application system (Nexcelom Bioscience, Lawrence, MA, USA) for 5 days. The cell proliferation rate was calculated as the fold change in cell number per unit time (days) based on the day 1 value.

### Cell Cycle and Apoptosis Assays

For cell cycle analysis, cells were harvested and stained with propidium iodide after pretreatment with RNase A. Guava easyCyte HT (Millipore, Billerica, MA, USA) was used for the cytometry experiments, and FlowJo software (version 10.0.7) was used to analyze the cell cycle and apoptosis. The Caspase-Glo VR3/7 Kit (Promega) was used for cell apoptosis detection, according to the manufacturer's instructions. Briefly, cells were plated into 96-well plates at a density of 1.0 × 10^4^/well in 100 μL of complete medium. Then, 100 μL of Caspase-Glo3/7 Reagent was added to each well and mixed on a plate shaker at 300 rpm for 30 s. Next, the cells were further incubated at room temperature for 1–2 h, and the optical density was measured using a plate reader (Tecan).

### Cell Invasion and Migration Assays

Cell migration and invasion assays were performed using chambers containing a polyethylene terephthalate membrane (Corning Inc., Corning, NY, USA). Briefly, 1 × 10^5^ cells were suspended in serum-free medium and added to the upper chamber, which was coated with (invasion assay) or without (migration assay) Matrigel. Complete medium containing 20% fetal calf serum was placed in the lower chamber. After culturing for 24 h (migration assay) or 40 h (invasion assay), the migrated or invaded cells at the bottom of the membrane were fixed, stained, and counted from nine random fields under an inverted microscope (200 × magnification, Nikon Corporation, Tokyo, Japan), respectively.

### Scratch Assay

Cells were seeded into 96-well culture plates at an appropriate density to reach over 90% cell confluence by the next day. The supernatants were then renewed with medium containing 1% FBS. A straight line was drawn on the surface of the cell monolayer with a 96-well wounding replicator (VP Scientific, San Diego, CA, USA) by aligning the center of the lower end of the 96-well plate and pushing upward to form a scratch. The cells expressing fluorescent protein were imaged in a dark field, and the area of confluent cells was counted using the Celigo Cell Counting application system (Nexcelom Bioscience) at 0, 6, and 24 h. The increase of confluence was directly used to measure cell migration with the formula: MRt = [(StL + StR)–(S0L + S0R)]/[1–(S0L + S0R)], where MRt is the migration rate at hour t, StL, and StR are the areas at the left and right sides of the scratch line at hour t, respectively, and S0L and S0R are the areas at the left and right sides of the scratch line at hour 0, respectively.

### Statistical Analysis

Statistical analyses were performed using IBM SPSS 23.0 (IBM Corp, Armonk, NY, USA). Data are expressed as the mean ± standard deviation (medians, ranges) and frequencies, as appropriate. The Student's *t-*test (or the Wilcoxon signed-rank test, when appropriate) was used to compare continuous variables, and the chi-squared test (or Fisher's exact test, when appropriate) was used to compare categorical variables. The repeated measures analysis of variance test was used to compare the multi-time point measurement variables. The Kaplan–Meier method was used to investigate survival differences between groups, which were examined with the aid of the log-rank test. Two-sided *P* < 0.05 indicated a significant difference.

## Results

### DDX39 Overexpression Is Correlated With Progressive Clinicopathological Features and a Poor Prognosis of Melanoma Patients

**Figure 1:**
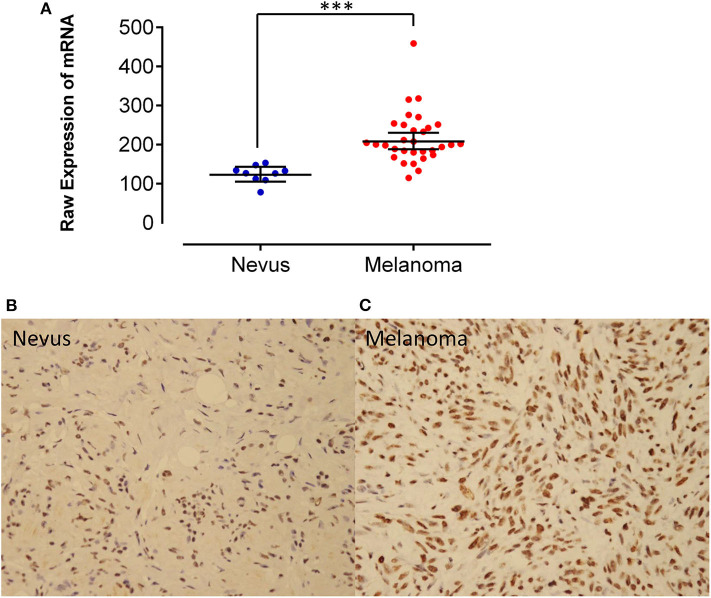
**(A)** By analyzing the Affymetrix expression profile in the GEO database GSE 46517, DDX39, a member of the DEAD box RNA helicase family, was identified as an overexpressed gene in melanoma but not in pigmented nevi tissues. Immunohistochemical staining of DDX39 was weak in pigmented nevus tissues **(B)**, while it was stronger in human melanoma tissues **(C)**. Magnification, 400×. ***P < 0.001.

**Figure 2:**
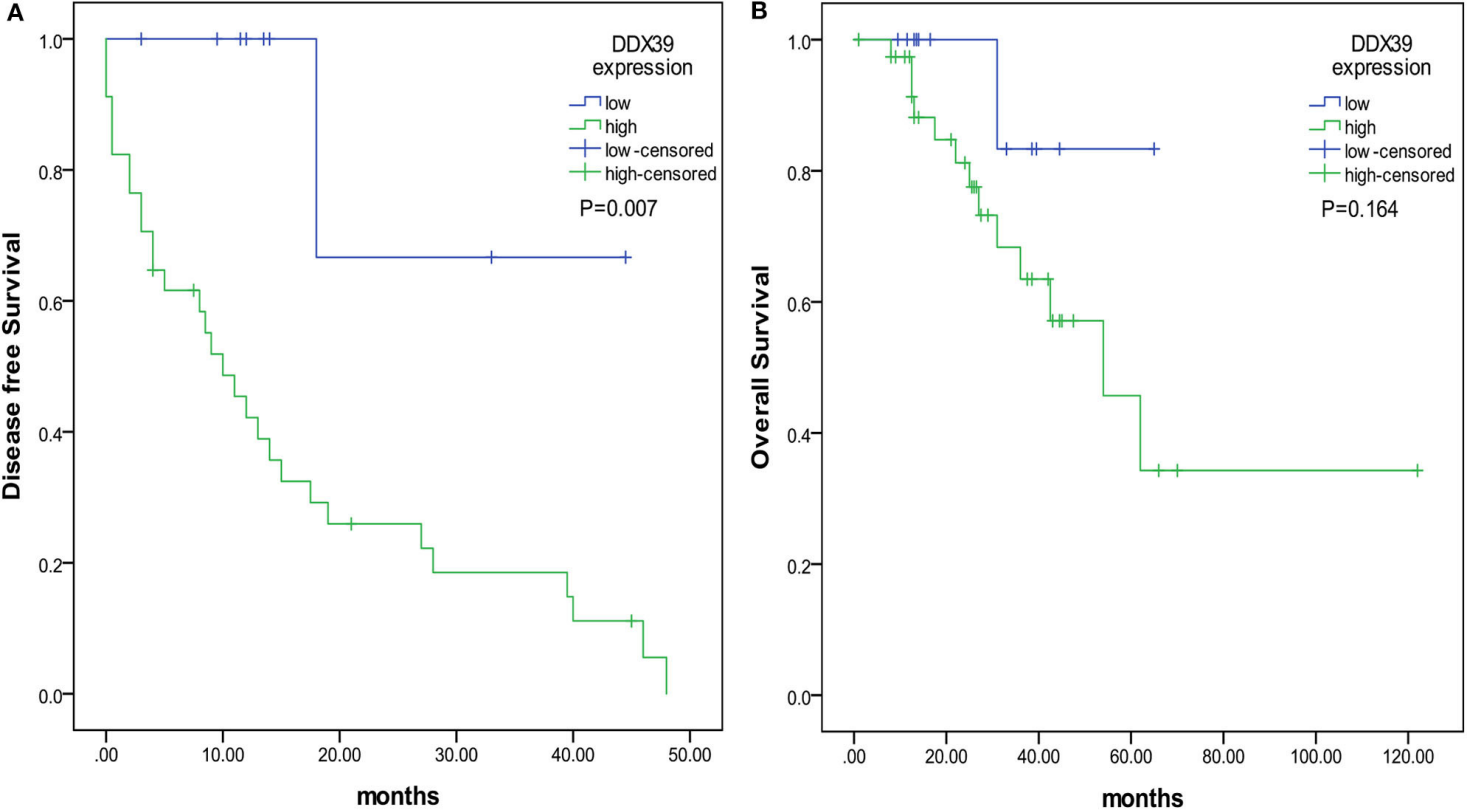
The prognostic value of DDX39 overexpression in melanoma patients. The overall survival time was counted from the day of operation to the end of follow-up. The disease-free survival time was counted from the date of surgery to the time of disease recurrence or metastasis. **(A)** Patients with a higher expression of DDX39 had a shorter disease-free survival time. **(B)** Patients with a higher expression of DDX39 also had a shorter overall survival time.

By analyzing the Affymetrix expression profile in the GEO database GSE 46517, we identified DDX39, a member of the DEAD box RNA helicase family, as an overexpressed gene in melanoma but not in pigmented nevi tissues (Figure 1A; *P* < 0.001). As shown in Figures 1B,C, immunohistochemical analysis revealed that DDX39 protein is mainly found in the nucleus, although the cytoplasm contains a small amount of it. The expression of DDX39 protein was significantly higher in melanoma (Figure 1C) than in pigmented nevus tissue (Figure 1B, Table 1; *P* < 0.001). Further analysis of 15 cases of melanoma and their matched recurrent and metastatic tissues showed that DDX39 was highly upregulated in the recurrent and metastatic tissues (Table 2; *P* = 0.016). The overexpression of DDX39 protein was also positively correlated with the American Joint Committee on Cancer (AJCC) clinical stage (*P* = 0.014), Breslow thickness (*P* = 0.014), and Clark grade (*P* = 0.009) (Table 3). As shown in Figure 2B, the overall survival time was shorter in patients with an elevated expression of DDX39 compared to patients with a lower expression of DDX39, although the difference was not statistically significant. However, patients with a higher DDX39 expression had a poorer prognosis, as characterized by a significantly shortened disease-free survival time (Figure 2A) (*P* = 0.007). Taken together, these data suggest that DDX39 may play an important role in promoting melanoma aggressiveness.

**Table 1:** DDX39 expression in melanoma and pigmented nevi patients.

		**DDX39 expression**, ***n*** **(%)**		
	***n***	**High expression**	**Low expression**	***P*-value**
Melanoma	51	39 (78.2%)	12 (21.8%)	<0.001
Nevus	10	1 (10.0%)	9 (90%)	

*Data were analyzed by Fisher's exact test. P < 0.001*.

**Table 2:** The expression of DDX39 protein in metastatic and recurrent melanoma was stronger than that in primary melanoma.

	**DDX39 expression**	***P*-value**
	**Median (25, 75%)**	
Primary melanoma	270 (260, 280)	0.016^**^
Recurrent/metastatic melanoma	300 (300, 300)	

*Data were analyzed by the Wilcoxon signed-rank test*,

** *P < 0.01*.

**Table 3:** Correlation between DDX39 expression and clinicopathological factors in 51 patients with melanoma.

**Clinicopathological factor**	**DDX39 expression**		
	**High, *n* (%)**	**Low, *n* (%)**	***P*-value**
Age			
≤ 60	11 (68.8%)	5 (31.3%)	0.601
>60	28 (80.0%)	7 (20.0%)	
Sex			0.027^*^
Male	24 (88.9%)	3 (11.1%)	
Female	15 (62.5%)	9 (37.5)	
Breslow depth			0.014^*^
≤ 2 mm	9 (52.9%)	8 (47.1%)	
>2 mm	30 (88.2%)	4 (11.8%)	
AJCC stage			0.014^*^
0–IIA	9 (52.9%)	8 (47.1%)	
IIB–IV	30 (88.2%)	4 (11.8%)	
Ulceration			0.741
Present	33 (78.6%)	9 (21.4%)	
Absent	6 (66.7%)	3 (33.3%)	
Clark level			0.009^**^
1–3	6 (42.6%)	7 (53.8%)	
4–5	33 (86.8%)	5 (13.2%)	
Pathological type			0.016^*^
Superficial spreading subtype	17 (63.0%)	10 (37.0%)	
Nodular subtype	22 (91.7%)	2 (8.3%)	
Lymphovascular invasion			0.315
Yes	6 (100%)	0 (0.0%)	
No	33 (73.3%)	12 (26.7%)	
Nerve aggression			1.000
Yes	5 (71.4%)	2 (28.6%)	
No	34 (77.3%)	10 (22.7%)	
Lymphocyte infiltration			0.294
Yes	11 (64.7%)	6 (35.3%)	
No	28 (82.4%)	6 (17.6%)	
Tumor site			0.801
Acem-terminal	29 (74.4%)	10 (25.6%)	
Non-Acem-terminal	10 (83.3%)	2 (16.7%)	
Mitosis (n/mm^2^)			0.851
≤ 5	26 (74.3%)	9 (25.7%)	
>5	13 (81.3%)	3 (18.8%)	

*Data were analyzed by the chi-squared text or Fisher's exact test*,

* *P < 0.05*,

** *P < 0.01*.

### Downregulation of DDX39 Protein Impaired the Proliferation of Melanoma Cells

To examine the above *ex vivo* discoveries, a series of *in vitro* experiments were conducted. The impact of DDX39 downregulation on the proliferation, invasion, and migration of melanoma cells was investigated by RNA interference mediated by lentivirus. Both A375 and SKMEL28 melanoma cells were transduced with a lentivirus expressing DDX39 RNAi (sh*DDX39* group) or a negative control lentivirus (shCtrl group). As indicated in Figure S1, the results of qPCR (A and B) and western blotting (C and D) indicated that shDDX39 but not shCtrl effectively reduced the expression of DDX39 in both A375 and SKMEL28 cells.

**Figure 3:**
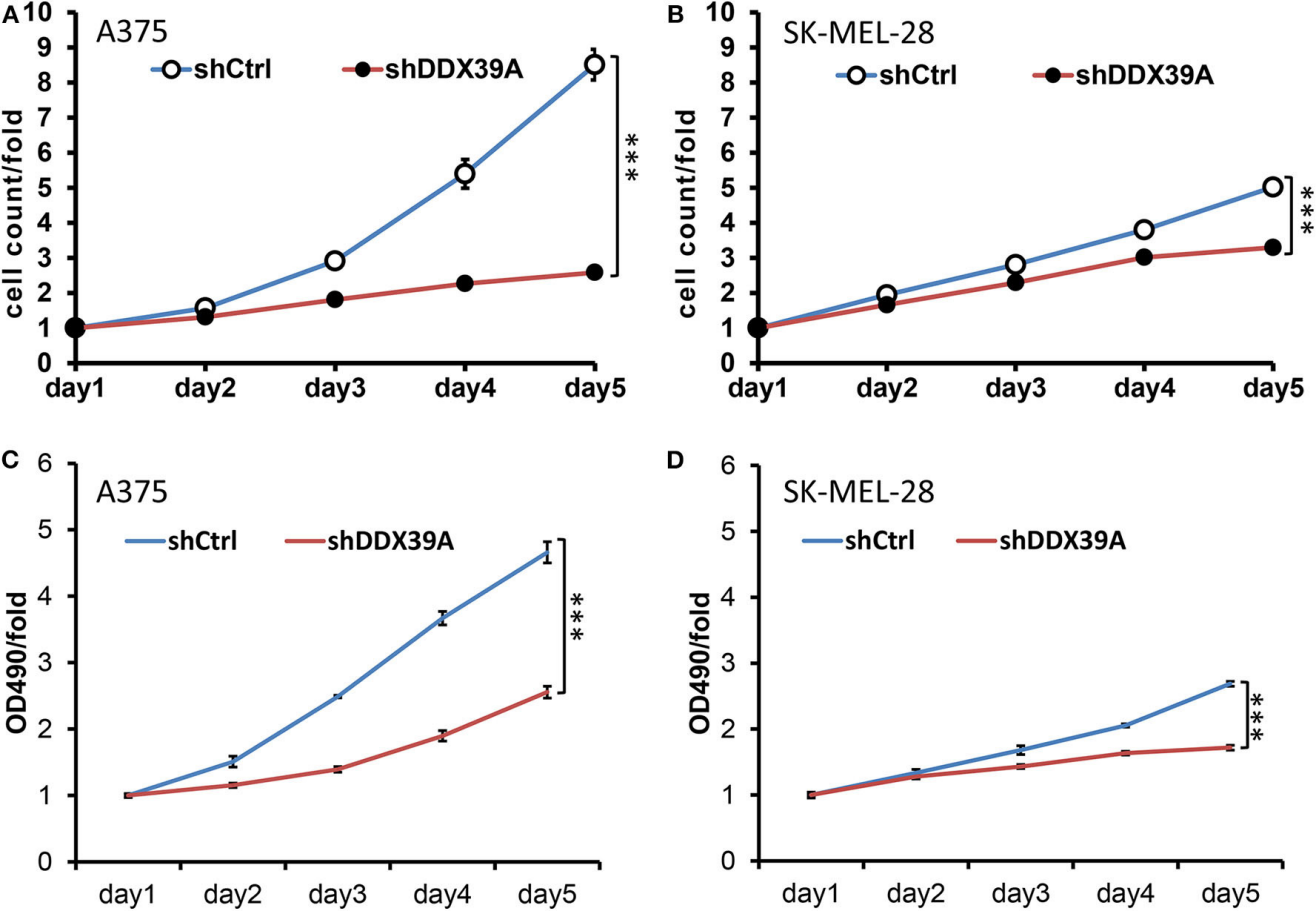
The impact of DDX39 downregulation on the proliferation, invasion, and migration of melanoma cells was investigated by RNA interference mediated by lentivirus. The effect of DDX39A knockdown on the cell proliferation of A375 **(A)** and SKMEL28 **(B)** cells was measured by the Celigo Cell Counting application system. Beginning on day 2, the cells were imaged and counted daily for 5 days. The effect of DDX39A knockdown on the cell growth of A375 **(C)** and SK-MEL28 **(D)** cells was assessed by the MTT experiment in triplicate daily for 5 days. The mean and standard deviation (SD) values of at least three repeated experiments are reported. ***P < 0.001, compared with the shCtrl group.

**Figure 4:**
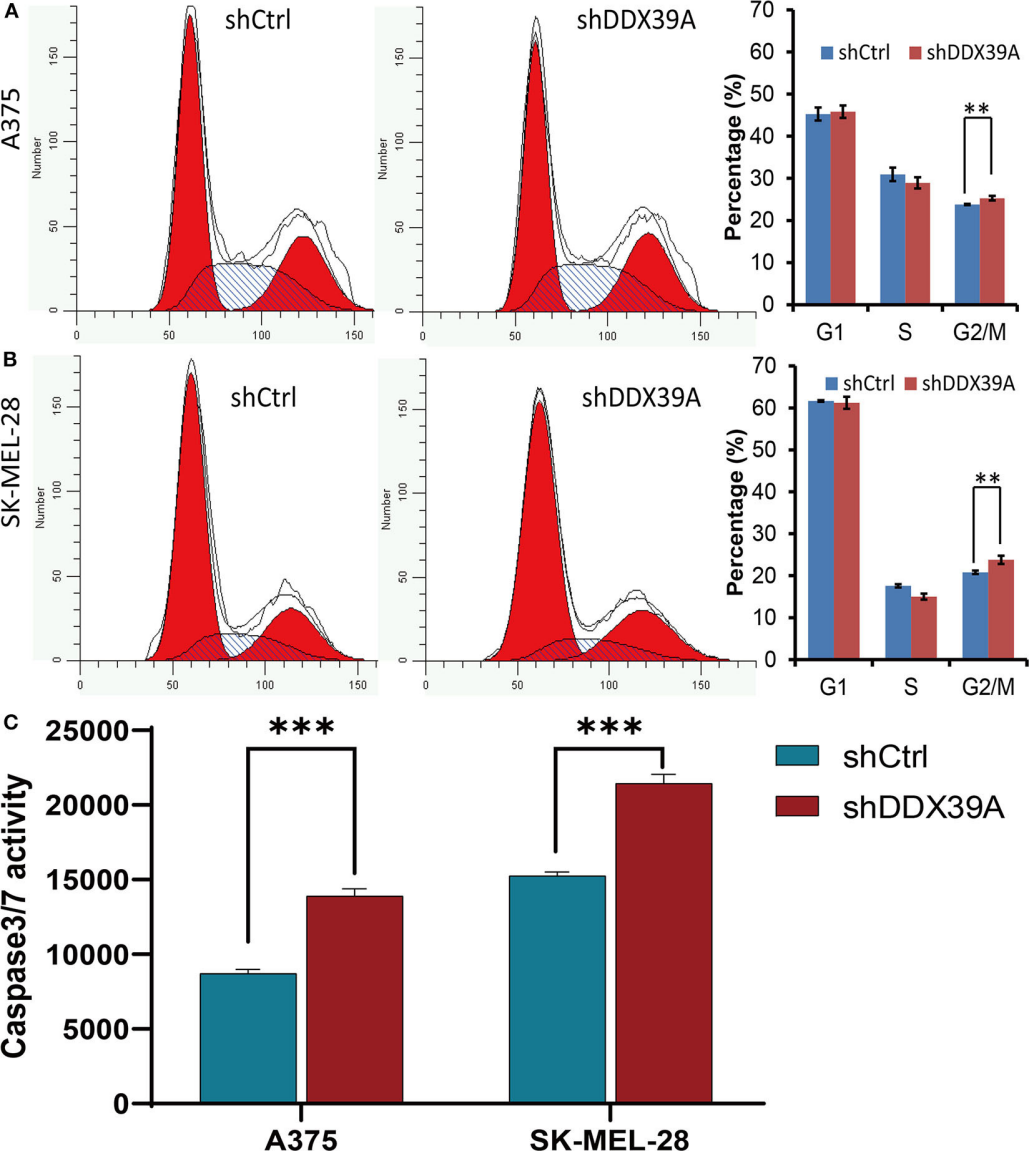
The effect of DDX39A knockdown on the cell cycle and apoptosis of melanoma cells was investigated by flow cytometry and the Caspase-Glo VR3/7 Kit assay. Compared with the shCtrl group, the numbers of A375 **(A)** as well as SK-MEL28 **(B)** cells in the G2 and M phases were increased in the shDDX39A group. **(C)** Compared with the shCtrl group, the caspase-3/7 activities of A375 and SKMEL28 cells were increased in the shDDX39A group. **P < 0.01, ***P < 0.001. The mean and standard deviation (SD) values of at least three repeated experiments are reported.

As shown in Figure 3A, the high-content screening cell count experiment demonstrated that the fold change of the A375 cell number was significantly lower in the shDDX39A group (2.58 ± 0.05) than in the shCtrl group (8.51 ± 0.44) (*P* < 0.001) from day 1 to 5. Similarly, the fold change of the SKMEL28 cell number was significantly lower in the shDDX39A group (3.30 ± 0.04) than in the shCtrl group (5.02 ± 0.14) (*P* < 0.001) (Figure 3B). Consistently, the MTT assay showed that the fold change of the A375 cell number in the shDDX39A group (2.56 ± 0.09) was significantly lower compared to that in the shCtrl group (4.66 ± 0.16) (*P* < 0.001) (Figure 3C). The fold change of the SKMEL28 cell number was significantly lower in the shDDX39A group (1.72 ± 0.04) than in the shCtrl group (2.69 ± 0.04) (*P* < 0.001) (Figure 3D). In addition, cell cycle analysis showed that after 5 days of cell culture, the proportion of A375 cells in the G2/M phase of the shCtrl group was 23.79 ± 0.14%, which was significantly lower than that of the shDDX39A group (25.28 ± 0.54%) (*P* = 0.009) (Figure 4A). For SKMEL28 cells, the proportion of G2/M cells in the shCtrl group was 20.79 ± 0.41%, which was lower than that of the shDDX39A group (23.78 ± 0.97%) (*P* = 0.007) (Figure 4B). As shown in Figure 4C, after culturing A375 cells for 5 days, the fluorescence intensity value of the caspase-3/7 activity in the shCtrl group (8727.33 ± 254.58) was significantly lower than that in the shDDX39A group (13902.00 ± 486.72) (*P* < 0.001). For SKMEL28 cells, the fluorescence intensity value of caspase-3/7 activity in the shCtrl group was 15260.30 ± 259.50, which was significantly lower than that in the shDDX39A group (21447.30 ± 605.48) (*P* < 0.001). Taken together, these results indicate that RNA interference against DDX39 can suppress melanoma cell growth *in vitro* by enhanced caspase-mediated apoptosis or increased G2/M cell cycle arrest, but the role of the latter may not be as significant as the former.

### Downregulation of DDX39 Protein Impaired the Invasion and Migration of Melanoma Cells

**Figure 5:**
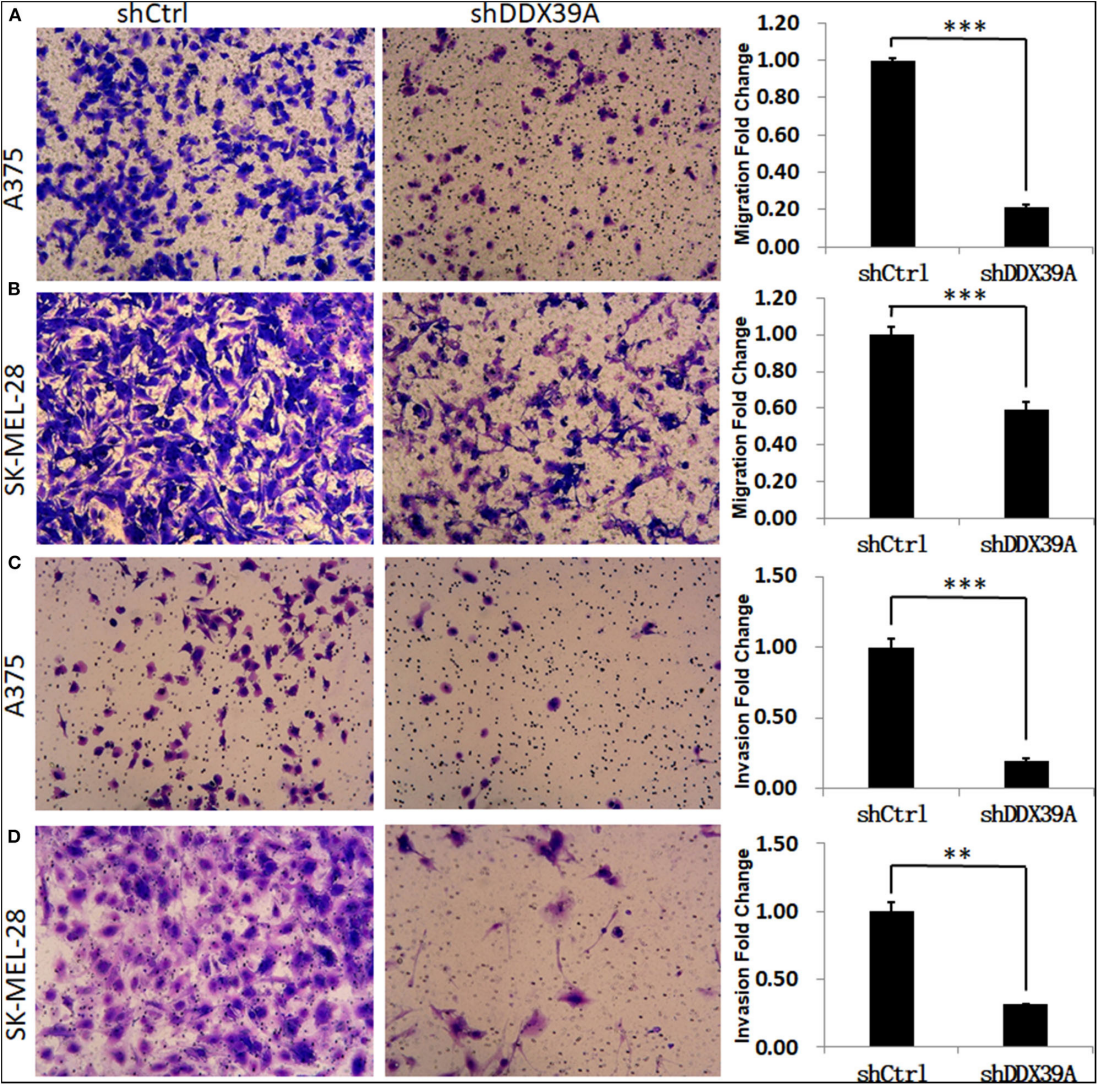
The effect of DDX39A knockdown on the migration and invasion of melanoma cells. Compared with the shCtrl group, the transwell chamber assay showed that the migration **(A,B)** and invasion **(C,D)** abilities were obviously inhibited in shDDX39A-modified A375 **(A,C)** and SK-MEL28 **(B,D)** cells. Magnification, 100×. **P < 0.01, ***P < 0.001. The mean and standard deviation (SD) values of at least three repeated experiments are reported.

**Figure 6:**
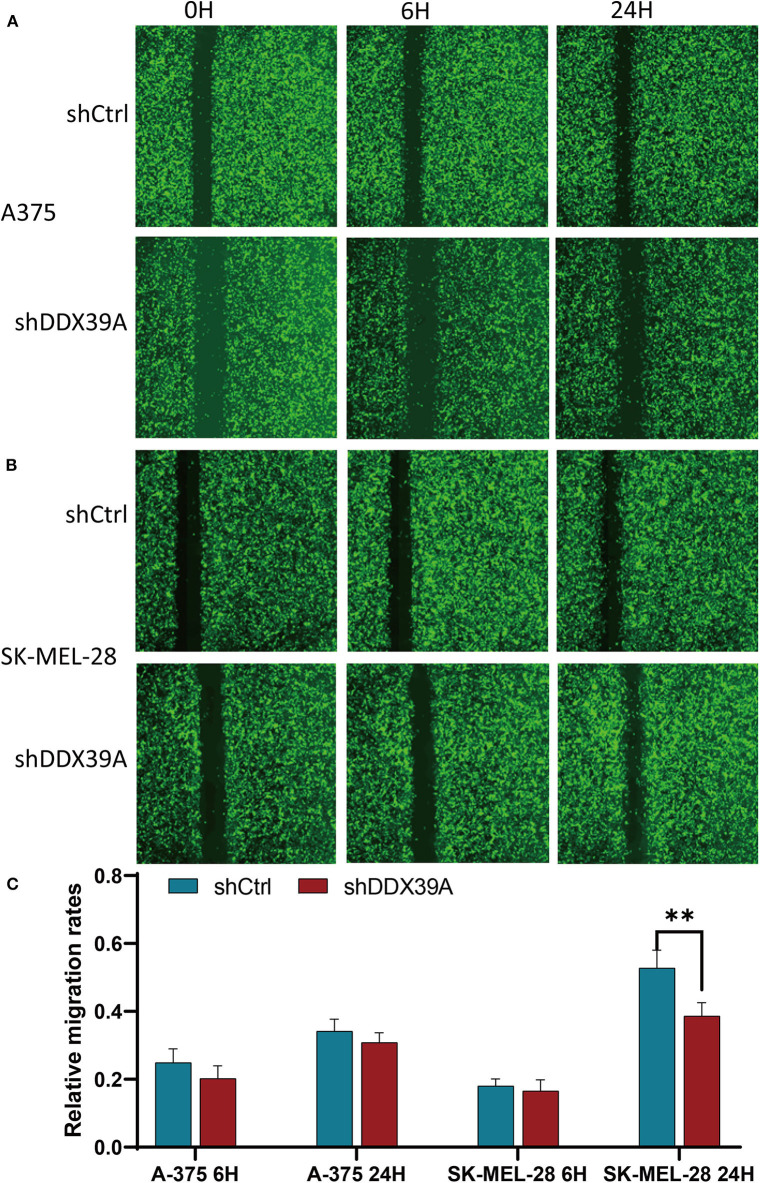
The effect of DDX39A knockdown on the wound healing of melanoma cells. The scratch width and the relative migration rates of A375 **(A,C)** and SK-MEL28 **(B,C)** cells in the shDDX39A group and the shCtrl group were imaged and counted using the Celigo Cell Counting application system at 0, 6, and 24 h, separately. **P < 0.01. The mean and standard deviation (SD) values of at least three repeated experiments are reported.

The transwell migration experiment showed that more A375 cells transferred to the lower chamber in the shCtrl group (121 ± 1.29) than in the shDDX39A group (26 ± 1.39) (*P* < 0.001) (Figure 5A). The number of SK-MEL-28 cells found in the lower chamber in the shCtrl group (131 ± 5.42) was also much more than that in the shDDX39A group (78 ± 5.27) (*P* < 0.001) (Figure 5C). The invasion test showed that the number of A375 cells invaded to the lower chamber in the shCtrl group was 37 ± 2.27, which is significantly higher than that in the shDDX39A group (7 ± 0.5) (*P* < 0.001) (Figure 5B). Similarly, ~62 ± 4.35 invaded SK-MEL-28 cells/well were observed in the shCtrl group, whereas only 19 ± 0.22 cells/well were observed in the shDDX39A group (*P* = 0.003) (Figure 5D). The scratch test results showed that after 6 h, the migration rate of SK-MEL-28 cells in the shDDX39A group (16.53 ± 3.27%) was lower than that of the shCtrl group (18.03 ± 2.07%) (Figures 6B,C), although the difference was not statistically significant (*P* = 0.413). However, after 24 h, the migration rate of SK-MEL-28 cells in the shDDX39A group (38.61 ± 3.98%) was significantly lower than that of the shCtrl group (52.80 ± 5.22%) (*P* = 0.001) (Figures 6B,C). As shown in Figures 6A,C, the migration rate of A375 cells after 6 h in the shDDX39A group (20.20 ± 3.79%) was lower than that of the shCtrl group (24.91 ± 3.79%), and the migration rate of A375 cells after 24 h in the shDDX39A group (30.81 ± 2.90%) was lower than that of the shCtrl group (34.19 ± 3.48%) (Figures 6A,C), although neither of the differences was statistically significant (*P* = 0.094 and *P* = 0.133, respectively). Our results revealed that the functional disturbance of DDX39 resulted in a significantly impaired melanoma cell invasion and migration ability *in vitro*, suggesting that DDX39 might be indispensable for tumor aggressiveness *in vivo*.

### Expression of SNAIL Was Downregulated by shDDX39

**Figure 7:**
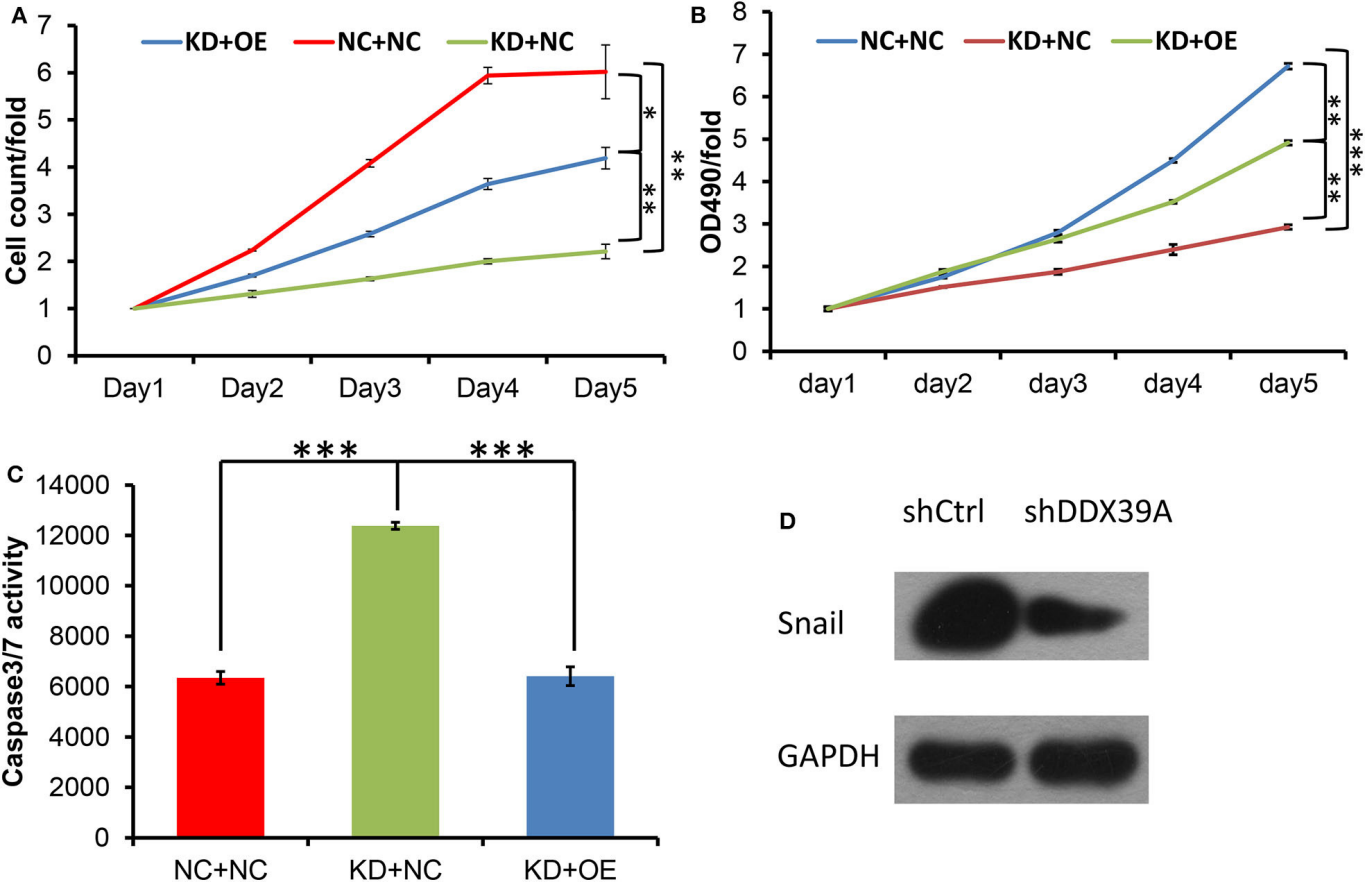
Both the high-content screening **(A)** and MTT **(B)** experiments showed that after the overexpression of SNAIL protein, the A375 cell proliferation rate was increased in the KD+OE group compared to the KD+NC group. **(C)** Compared with the KD+NC group, the caspase-3/7 activity of apoptotic A375 cells was decreased in the KD+OE group. **(D)** SNAIL was found to be downregulated in the shDDX39A group compared to the shCtrl group. *P < 0.05, **P < 0.01, ***P < 0.001. All analyses shown in this figure were calculated from three distinct experiments.

Previous studies have reported that SNAIL, one of the target genes of the Wnt/β-catenin pathway, can be positively regulated by DDX39 in hepatocellular carcinoma (9). SNAIL also has been found to be upregulated in melanoma (7). In order to identify whether SNAIL protein can possibly be regulated by DDX39 in melanoma, the differences in the expression of SNAIL between the shDDX39A and shCtrl groups were examined by western blot. As shown in Figure 7D, SNAIL was found to be downregulated in the shDDX39A group compared to the shCtrl group. It is reasonable to suspect that SNAIL, which was downregulated by the functional disturbance of DDX39, is more likely to be positively correlated with the melanoma aggressiveness induced by DDX39. Therefore, the overexpression of SNAIL may restore some of the DDX39 functions lost in shDDX39-modified melanoma cells. To prove this reasoning, recombinant lentivirus carrying the overexpression cassette of SNAIL and its control vector were introduced into A375 cells in the shDDX39A and shCtrl groups, respectively. As shown by the high-content screening cell proliferation results (Figure 7A), the fold change of the A375 cell number at day 5 was significantly decreased in the mock-transduced shDDX39A group (referred to as KD+NC hereinafter) compared with the mock-transduced shCtrl group (referred to as NC+NC hereinafter) (2.21 ± 0.15 vs. 6.02 ± 0.57, *P* = 0.005). In contrast, the A375 cells grew faster in the SNAIL-overexpressed shDDX39A group (referred to as KD+OE hereinafter) than in the KD+NC group (4.19 ± 0.23 vs. 2.21 ± 0.15, *P* = 0.009), suggesting that SNAIL is one of the effectors activated by DDX39.

### SNAIL Overexpression Promoted the Proliferation, Invasion, and Migration of Melanoma Cells Modified by shDDX39

**Figure 8:**
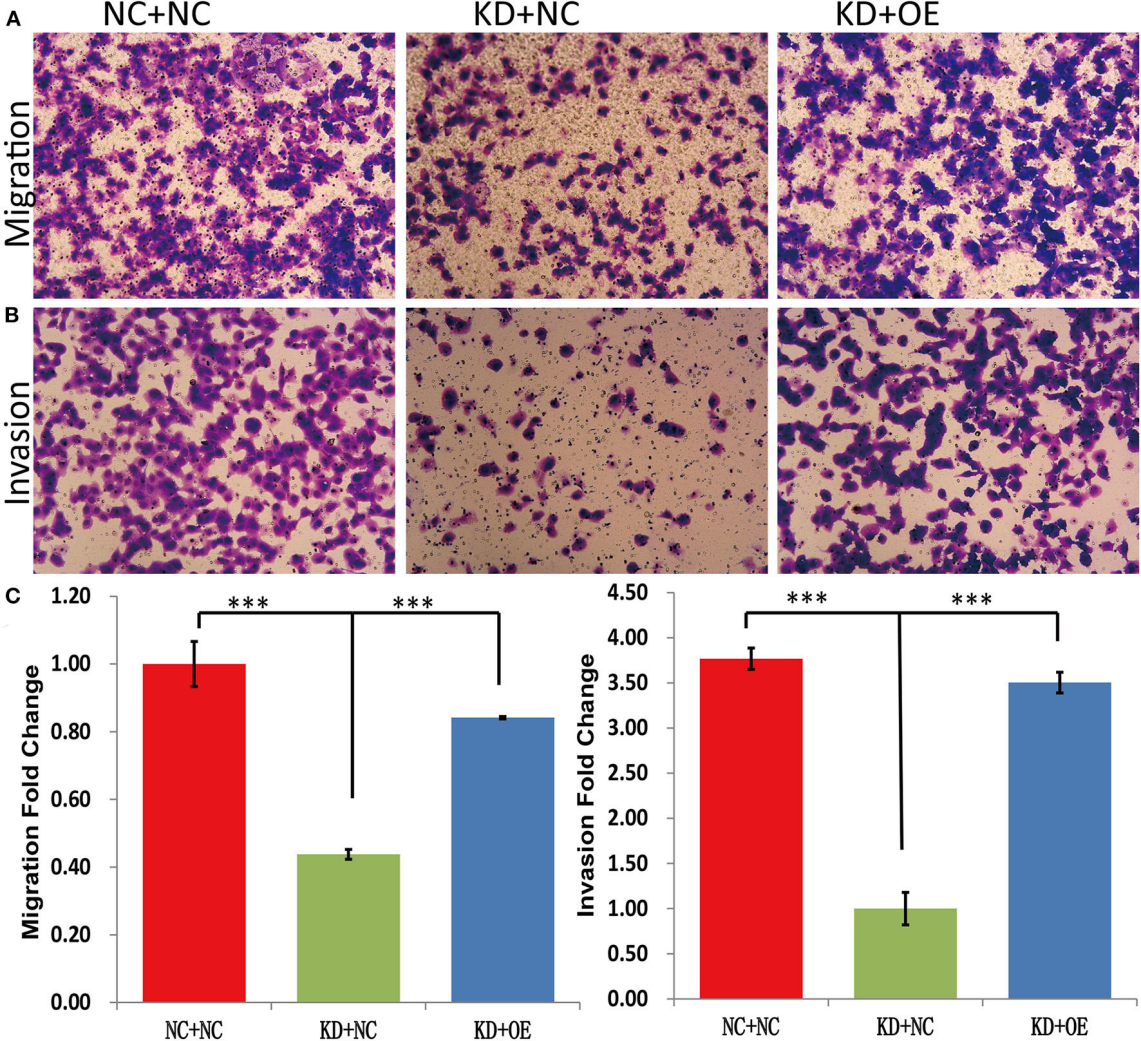
Transwell migration experiments **(A,C)** showed that the numbers of cells detected in the lower chamber in the NC+NC group, KD+NC group, and KD+OE group were 109 ± 7.22, 40 ± 1.60, and 91 ± 0.39, respectively, indicating that the migration of DDX39-knockdown melanoma cells can be promoted by SNAIL overexpression. Transwell invasion experiments **(B,C)** showed that the numbers of cells that moved to the lower chamber in the NC+NC group, KD+NC group, and KD+OE group were 113 ± 3.56, 30 ± 5.41, and 106 ± 3.46, respectively, indicating that the invasion of DDX39-knockdown melanoma cells can be activated by SNAIL overexpression. Magnification, 100×. ***P < 0.001. The mean and standard deviation (SD) values of at least three repeated experiments are reported.

The MTT results showed that the fold change of the A375 cell number at day 5 was significantly decreased in the KD+NC group compared with the NC+NC group (2.93 ± 0.05 vs. 6.72 ± 0.07, *P* < 0.001). In contrast, the A375 cells grew faster in the KD+OE group than in the KD+NC group (4.91 ± 0.05 vs. 2.93 ± 0.05, *P* = 0.001) (Figure 7B), indicating that the proliferation of DDX39-knockdown melanoma cells can be stimulated by SNAIL overexpression. The caspase-3/7 activity experiments showed that the apoptosis activities in the NC+NC group, KD+NC group, and KD+OE group were 6348 ± 248.6, 12381.67 ± 138.87, and 6408.67 ± 371.30, respectively (Figure 7C), indicating that the apoptosis in DDX39-knockdown melanoma cells can be inhibited by SNAIL overexpression (*P* < 0.001). As shown in Figure 8, the transwell migration experiments showed that the numbers of cells detected in the lower chamber in the NC+NC group, KD+NC group, and KD+OE group were 109 ± 7.22, 40 ± 1.60, and 91 ± 0.39, respectively, indicating that the migration of DDX39-knockdown melanoma cells can be promoted by SNAIL overexpression (*P* < 0.001). Transwell invasion experiments showed that the numbers of cells that moved to the lower chamber in the NC+NC group, KD+NC group, and KD+OE group were 113 ± 3.56, 30 ± 5.41, and 106 ± 3.46, respectively, indicating that the invasion of DDX39-knockdown melanoma cells can be activated by SNAIL overexpression (*P* < 0.001). These data collectively suggest that SNAIL can play a role in the DDX39 pathway by activating the aggressiveness of melanoma cells.

### Overexpression of DDX39 and SNAIL Was Positively Correlated

Immunohistochemical staining of DDX39 and SNAIL was performed on 51 melanoma tissues. The results showed that there was a positive correlation between DDX39 overexpression and SNAIL overexpression in melanoma tissues, with an odds ratio of 6.67 (95% confidence interval: 1.62–27.38; *P* = 0.004; Table 4).

**Table 4:** The expression of DDX39 and SNAIL in human melanoma.

	**DDX39 expression**		
	**Low (0–200)**	**High (201–300)**	***P*-value**
SNAIL expression			0.004^**^
Low (–, +)	8	9	
High (++, +++)	4	30	

*Data were analyzed by Pearson's correlation test*.

** *P < 0.01*.

## Discussion

DDX39, the 49-kDa protein encoded by the gene DDX39A, is a member of the DEAD RNA helicase family, which includes DDX1, DDX3, DDX5, DDX17, and DDX39 (6). DEAD RNA helicase plays a key role in manipulation of the RNA structure. However, these proteins usually act as part of multi-protein complexes; therefore, their exact function depends heavily on other interacting partners. Hence, DEAD box members always have additional functions other than their known ATP-dependent RNA helicase activities. Numerous reports have indicated that DEAD box proteins are involved in processes that are key to cellular proliferation and/or neoplastic transformation, including the growth of tumor cells, cell cycle control, apoptosis, RNA translocation, cytoskeletal rearrangement, and cell migration (10–14).

The overexpression of DDX39 protein has recently been found in various human tumor tissues and cells, such as lung squamous cell carcinoma (15), gastrointestinal stromal tumors (16, 17), pancreatic cancer (18), prostate cancer (19, 20), hepatocellular carcinoma (9), and malignant mesothelioma (21). It also has been reported that DDX39 protein is downregulated in other tumors, such as bladder urothelial carcinoma infiltrating the muscle layer (22) and colorectal cancer (23), indicating that DDX39 protein may play either an oncogenic or tumor-suppressive role in different cancers. However, the role of DDX39 in melanoma development and progression is not well-understood.

By using GEO database analysis, we identified the DDX39A gene as a differentially expressed gene between melanoma and pigmented nevus tissue. We speculated that the upregulated DDX39 expression in melanoma and the lower expression in normal pigment cells or benign pigmented nevi are related to the biological function of DDX39. DDX39 is an RNA helicase, which mainly affects cellular processes such as RNA transcription and protein translation. In melanoma cells, upregulated expression of DDX39 is beneficial for a wide array of RNA activities, including RNA duplex unwinding, protein displacement from RNA, and strand annealing. In the present study, immunohistochemical examination revealed that the protein expression of DDX39 in melanoma tissue was significantly higher than that in pigmented nevus tissue. The overexpression of DDX39 was also positively correlated with the Breslow thickness, AJCC clinical stage, as well as metastasis and recurrence of melanoma. Consistently, DDX39 overexpression was correlated with a shorter progression-free survival time. In our *in vitro* study, DDX39 knockdown resulted in significantly suppressed proliferation, enhanced apoptosis, as well as decreased invasion and migration of melanoma cell lines. Taken together, these findings strongly support the idea that DDX39 expression is positively associated with melanoma aggressiveness.

In order to further identify the possible effectors regulated by DDX39, we found that SNAIL overexpression restored the proliferation, invasion, and migration of melanoma cells impaired by shDDX39 modification, suggesting that SNAIL is one of the effectors activated by DDX39. As a transcription factor, SNAIL also has been shown to promote the epithelial-mesenchymal transition and therefore regulate the development and progression of cancer. For example, SNAIL has been found to be able to inhibit the expression of cell adhesion proteins, such as E-cadherin, to accelerate tumor migration and invasion (24). Other studies have demonstrated that SNAIL can promote the occurrence of epithelial-mesenchymal transition via the Dock1/NF-kB/SNAIL pathway in breast cancer (25), via the AKT/GSK-3ß/SNAIL pathway in colon cancer (26), via the Eif4E/SNAIL pathway in nasopharyngeal carcinoma (27), and via the PI3K/AKT/SNAIL pathway in head and neck squamous cell carcinoma (28). SNAIL can also activate tumor cell proliferation by regulating CyclinD2, preventing cells from entering the end of the G1 stage, affecting proapoptotic factors, or inhibiting the tumor suppressor gene TP53 (29). In line with our results, a previous study has found that DDX3, a DDX39 homolog, exhibits an oncogenic effect by stabilizing SNAIL to reduce the expression of E-cadherin in HeLa and MCF-7 cells (30, 31). In another recent report, Zhang et al. (9) have found that the expression of target genes of the Wnt/β-catenin pathway, such as SNAIL, is upregulated by DDX39 hepatocellular overexpression, whereas it is downregulated by DDX39 knockdown in carcinoma cell lines. Our observations are also consistent with a previous study showing that SNAIL expression is elevated in melanoma (7).

To the best of our knowledge, this is the first systematic study to investigate the impact of DDX39 and SNAIL on melanoma aggressiveness both *in vitro* and *ex vivo*. We provided valuable evidence regarding the prognostic value of DDX39 for melanoma patients. Our findings also suggest that targeting DDX39/SNAIL may provide a novel strategy for melanoma therapy. Future studies will be worthwhile to determine the underlying mechanisms by which DDX39 controls the aggressiveness of melanoma and how it interacts with effectors, such as SNAIL.

## Data Availability Statement

The raw data supporting the conclusions of this article will be made available by the authors, without undue reservation.

## Ethics Statement

The studies involving human participants were reviewed and approved by Ethics Committee of the Second Affiliated Hospital of Dalian Medical University. The patients/participants provided their written informed consent to participate in this study.

## Author Contributions

LW, CX, and HT conceived and designed research. CX, HT, YZ, YT, QW, and LL collected data and conducted research. HT and KG analyzed and interpreted data. CX wrote the initial paper. LW revised the paper and had primary responsibility for the final content. All authors read and approved the final manuscript.

## Conflict of Interest

The authors declare that the research was conducted in the absence of any commercial or financial relationships that could be construed as a potential conflict of interest.

## Supplementary Material

Figure S1 Real-time RT-PCR and western blotting were used to evaluate the DDX39A expression at the mRNA and protein levels, respectively. Compared with the shCtrl group, both the mRNA and protein levels of DDX39A were significantly lower in the shDDX39A group. **(A)** Compared with the shCtrl group, the DDX39A mRNA level in A375 cells was significantly lower in the shDDX39A group. **(B)** Compared with the shCtrl group, the DDX39A mRNA level in SKMEL28 cells was significantly lower in the shDDX39A group. **(C)** Compared with the shCtrl group, the protein level of DDX39A in A375 cells was significantly lower in the shDDX39A group. **(D)** Compared with the shCtrl group, the protein level of DDX39A in SKMEL28 cells was significantly lower in the shDDX39A group. ^**^*P* < 0.01.
