# Supplementary figures and images for: DDX39 Overexpression Predicts a Poor Prognosis and Promotes Aggressiveness of Melanoma by Cooperating With SNAIL
Source: Front Oncol. 2020 Aug 12;10:1261. doi: 10.3389/fonc.2020.01261 (PMC7435017; doi:10.3389/fonc.2020.01261)

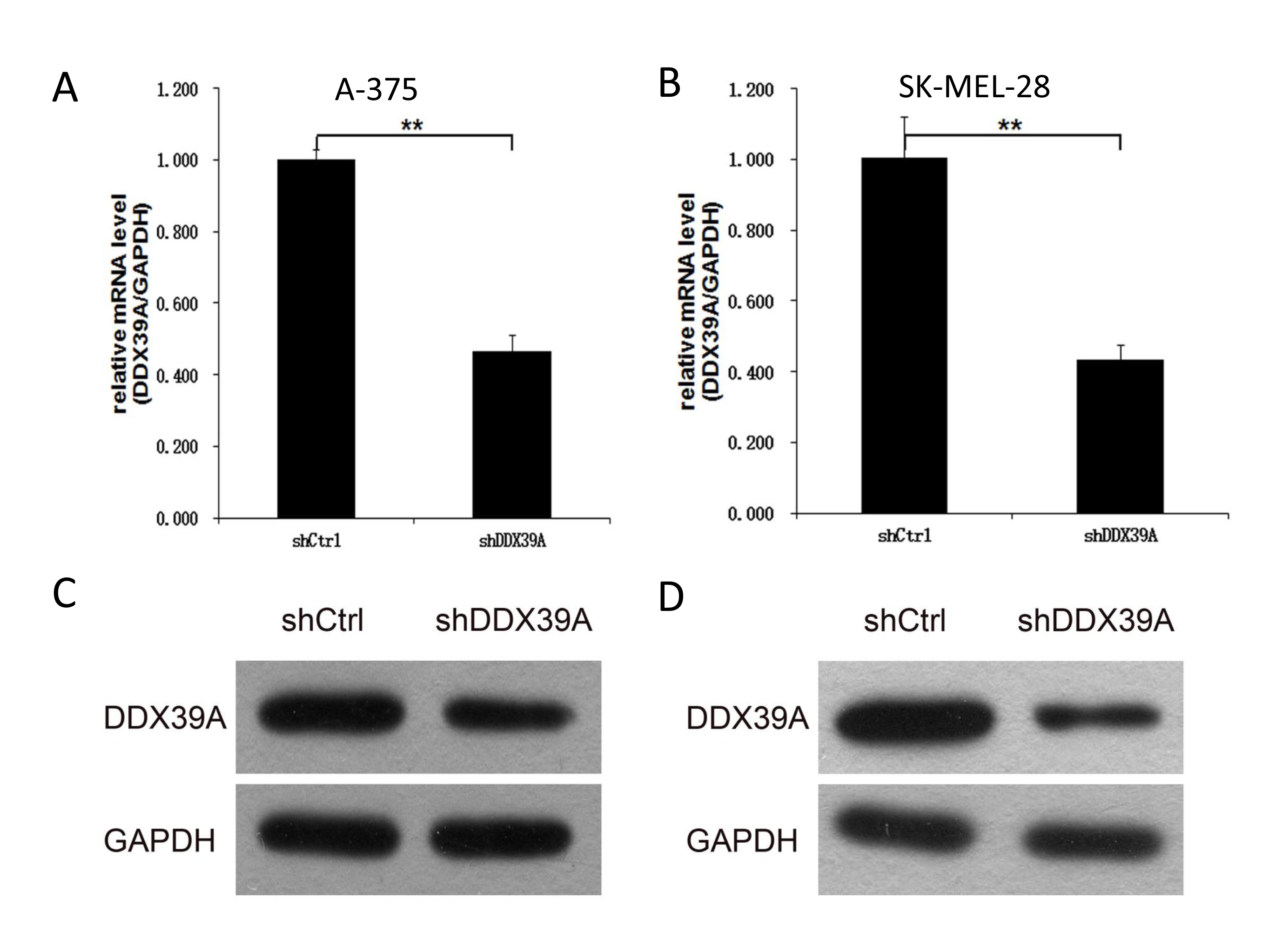

Supplement: Figure S1 — Real-time RT-PCR and western blotting were used to evaluate the DDX39A expression at the mRNA and protein levels, respectively. Compared with the shCtrl group, both the mRNA and protein levels of DDX39A were significantly lower in the shDDX39A group. (A) Compared with the shCtrl group, the DDX39A mRNA level in A375 cells was significantly lower in the shDDX39A group. (B) Compared with the shCtrl group, the DDX39A mRNA level in SKMEL28 cells was significantly lower in the shDDX39A group. (C) Compared with the shCtrl group, the protein level of DDX39A in A375 cells was significantly lower in the shDDX39A group. (D) Compared with the shCtrl group, the protein level of DDX39A in SKMEL28 cells was significantly lower in the shDDX39A group. **P < 0.01. [file Image_1.TIF]
